# News and Coming Events

**Published:** 1909-05-01

**Authors:** 


### NEWS AND COMING EVENTS.

THE Right Hon. Lord Viscount Duncannon has promised to preside at the festival dinner in aid of the funds of the Metropolitan Hospital at the Whitehall Rooms on May 20 next.

COLONEL ARTHUR SALTMARSHE, of Sevenoaks and Bournemouth, who died on March 11, left £500 to Gravesend Hospital and £500 to Guy's Hospital. Mrs. Esther Tate, of Princes Street, W., who died on March 11, left £1,000 to Middlesex Hospital.

MISS JUDITH HENDERSON, of Chester, left £600 to the Children's Convalescent Home, West Kirby, for the permanent endowment of a cot, and £500 to the Birkenhead and Wirral Children's Hospital, also for the endowment of a cot.

THE HON. HARRIET MARIA GEORGIANA LE POER TRENCH, who left estate valued at £122,769, bequeathed the net proceeds of the sale of her large diamond tiara and other valuable personal articles to the Hospital of St. Elizabeth of Hungary, Grove End Road, St. John's Wood, N.W., and on the falling in of certain annuities the capital of these is to be paid to this hospital, the testatrix's intention being that the bequest to the hospital shall amount to £10,000 from this source. She also left £100 each to the London Mendicity Society and nine other charitable institutions.

AT a special meeting of the members of the Cheshire Milk Producers' Association the following resolution was carried unanimously :—" That in view of the serious loss to stock-owners and to the nation resulting from bovine tuberculosis, this meeting is of opinion that the time has arrived when the question of a national scheme subsidised by Government for the insurance and subsequent elimination of all clinically diseased animals should receive the earnest consideration of the agricultural community and the Government."

MAJOR F. J. W. PORTER, R.A.M.C., appeals for funds in aid of the Princess Christian Mission Hospital at Sierra Leone, which was totally destroyed by fire on March 17. Though, thanks to the coolness and heroism of Miss MacPherson, one of the nursing sisters, no lives were lost, both the European and native sisters had the whole of their effects destroyed. The damage is partly covered by insurance, but money is immediately required to make it possible to carry on in a temporary building the excellent work which has hitherto been done by this hospital. It is also very desirable to reconstruct the building of stone, in order to minimise future risk of fire, and to build it on modern principles. Subscriptions may be sent to Mr. F. Fishwick, honorary treasurer, Sierra Leone Diocesan Fund, 20 Harold Road, Upper Norwood, S.E.

CANON ERSKINE CLARKE presided at the annual meeting of the governors of the Bolingbroke Hospital recently. In moving the adoption of the report he drew attention to the need for more subscriptions. Those for the year amounted to about £526. The cost of maintaining the accident branch of the hospital was £2,000 per annum. Donations and legacies had enabled all liabilities to be met, but the future was somewhat problematical. In seconding the adoption of the report, Dr. Howell said it was difficult to understand why the Wandsworth Borough Council (of which he is a member) should oppose the application of the Weir Bequest funds to the Bolingbroke Hospital. The number of cases from the Borough of Wandsworth treated at the hospital during the year was greater than that of those from Battersea.

MR. LYTTON P. MAITLAND, M.B., B.S. (Lond.), has been appointed Medical Registrar to Charing Cross Hospital.

DR. THOMAS CRAIG STEVENSON, medical officer of the Education Committee of the Somerset County Council, has been appointed Superintendent of Statistics of the General Register Office, Somerset House.

THE Therapeutical and Pharmacological Section of the Royal Society of Medicine will meet at 20 Hanover Square, W., on Tuesday, May 4, at 4.30 P.M., when Dr. Hale White and Dr. Eyre will open a discussion on the subject of " Vaccine Treatment."

THE British Balneological and Climatological Society will hold its annual provincial meeting this year at Torquay, the date being fixed for Saturday, May 8. As in previous years, arrangements are being made for the comfort and enjoyment of members attending the Conference.

LORD SANDHURST, treasurer of St. Bartholomew's Hospital, has received from Mr. Henry T. Butlin, D.C.L., F.R.C.S., consulting surgeon to the hospital, £100 towards the pathological block, being a third donation of that amount to the rebuilding fund of the hospital.

AT a provincial sessional meeting of the Royal Sanitary Institute, which will be held at the University of Birmingham on Saturday, May 8, a discussion on Tuberculosis and the Milk Supply will be opened at 11 A.M. by Mr. J. Malcolm, F.R.C.V.S., Veterinary Superintendent to the Birmingham Corporation.

AN appeal is made by the editor of the *Poultry World* for 20,000 new-laid eggs for distribution among the London hospitals. Working in conjunction with the Hospital Saturday Fund, he has arranged to receive at the offices of the *Poultry World* any contributions that may be made by poultry-keepers between May 17 and May 22. The eggs should be carefully packed and the carriage should be paid to the offices of the journal, at 154 Fleet Street, London, E.C. As the packages will be sent direct to the hospitals without being opened, it is requested that covering letters should be sent separately. No eggs should be sent before May 17.

AT the annual meeting of the Metropolitan Provident Medical Association, held at the offices, Lamb's Conduit Street, the report of the Council, which was adopted, showed that despite generally unfavourable conditions, the past year was on the whole satisfactory. The twenty branches—sixteen dispensaries and four medical clubs—have now a membership roll of 12,040 cards, family and single. The receipts from members were £5,197 18s. 3d. The medical officers received £3,314 16s. 6d., making, with the sum expended on dispensaries and drugs, a total of £4,946 15s. 3d. devoted to the medical part of the work, or 89 per cent. of the income. The Council call special attention to the action taken by the Education Committee of the London County Council with regard to the medical treatment of school children; and also to the Poor Law Commission's Report. The Council maintains that unless the dispensaries are prepared to make some modification of their present rules to meet the needs of the 750,000 children of school age, school clinics under the immediate management of the local education authority must sooner or later be established. This prospect they view with great misgiving, and they feel that it is their duty to make every effort to prevent a further relaxation of the sense of parental responsibility.

